# Magnetic stimulation for stress urinary incontinence: study protocol for a randomized controlled trial

**DOI:** 10.1186/s13063-015-0803-1

**Published:** 2015-06-21

**Authors:** Renly Lim, Men Long Liong, Wing Seng Leong, Nurzalina Abdul Karim Khan, Kah Hay Yuen

**Affiliations:** School of Pharmaceutical Sciences, Universiti Sains Malaysia, 11800 Pulau Pinang, Malaysia; Department of Urology, Island Hospital, Penang, Malaysia; Department of Urology, Lam Wah Ee Hospital, Penang, Malaysia

**Keywords:** Magnetic stimulation, Randomized controlled trial, Stress urinary incontinence, Study protocol

## Abstract

**Background:**

There is currently a lack of randomized, sham-controlled trials that are adequately powered, using validated outcomes, to allow for firm recommendations on the use of magnetic stimulation for stress urinary incontinence. We report a protocol of a multicenter, randomized, double-blind, sham-controlled parallel-group trial to evaluate the efficacy of magnetic stimulation for stress urinary incontinence.

**Methods/Design:**

One hundred twenty subjects with stress urinary incontinence will be randomized in a 1:1 allocation to either active or sham magnetic stimulation using computer-generated, permuted blocks of variable sizes. Subjects will receive 2 sessions of magnetic stimulation per week for 8 weeks (16 sessions total). The primary outcome is the improvement in severity of involuntary urine loss based on the International Consultation on Incontinence Questionnaire for Urinary Incontinence Short Form at the end of treatment sessions compared with baseline. Secondary outcomes include cure, stress urinary incontinence–related symptoms (incontinence episode frequency, urine loss in 1-hour pad test, pelvic floor muscle strength) and health-related quality of life (Patient Global Impression of Improvement, International Consultation on Incontinence Questionnaire–Lower Urinary Tract Symptoms Quality of Life and EQ-5D). The safety of magnetic stimulation will also be assessed. Besides evaluation of clinical treatment effectiveness, cost-effectiveness analysis using patient-reported outcomes will be performed.

**Discussion:**

This trial is designed to provide pending outcome information on this non-invasive treatment option. We intend to acknowledge the existing flaws in previous clinical trials and determine conclusively whether magnetic stimulation is effective for stress urinary incontinence.

**Trial registration:**

ClinicalTrials.gov Identifier: NCT01924728. Date of Registration: 14 August 2013.

**Electronic supplementary material:**

The online version of this article (doi:10.1186/s13063-015-0803-1) contains supplementary material, which is available to authorized users.

## Background

Stress urinary incontinence (SUI) is a condition in which there is an involuntary loss of urine on effort, physical exertion, sneezing or coughing [1]. It is a chronic and debilitating condition that affects the physical, psychological, social and economic well-being of affected individuals and their families [2] and substantially reduces quality of life (QoL) similarly to severe chronic diseases such as stroke, arthritis and chronic kidney disease [3, 4]. Prevalence estimates of urinary incontinence (UI) are disparate, ranging from 7 % to 53 % [5, 6], depending on variables such as definition of UI used, survey population, methodological and cultural differences, with SUI accounting for approximately 50 % of all incontinence [2].

The current guideline advocates pelvic floor muscle training (PFMT) as the first-line conservative treatment for SUI [7]. Use of other conservative therapeutic options, such as biofeedback, vaginal cones, electrical stimulation and urethral plugs, is limited because of their side effects, discomfort and invasiveness. Although surgical interventions using midurethral slings have unquestionably superior cure rates of approximately 90 %, compared with 30 % in conventional physiotherapy [8], some patients may be medically unfit for surgery, especially elderly patients. Moreover, there is a risk of adverse events related to surgery, such as bladder perforation, vaginal epithelial perforation and hematoma [8]. Indeed, when given a choice, most patients opt for conservative treatments [9]. Treatment options should therefore be based on the collaborative efforts between patients and doctors, taking into account both the patient’s preferences and the surgeon’s judgment [10].

Given the high SUI prevalence and its overwhelming negative impact on the QoL of patients, there is a need for an acceptable therapeutic option with high efficacy. Many subsequent studies have focused on developing novel, non-invasive techniques to treat SUI, including the use of magnetic stimulation (MS). Several small studies, conducted in the United States [11, 12], Japan [13–15], Korea [16] and Turkey [17, 18], demonstrated that MS improved symptoms of SUI, with limited or no side effects. Additional file 1 provides a summary of published clinical trials on MS for SUI in more detail. We recently conducted a systematic review and found that most of the previous MS studies on SUI had flaws, including lack of a placebo group, small sample size, short or no follow-up and poor reporting [19]. The Fifth International Consultation on Incontinence emphasized that no recommendation is possible based on current evidence and that further research is needed to reduce the uncertainty around decision-making regarding the use of MS [20]. Many key issues remain. Is MS effective for female SUI? How many sessions are needed? How long can the effects last? Is maintenance therapy required? To answer these questions, a high-quality randomized controlled trial is imperative. Thus, we designed a multicenter, randomized, double-blind, sham-controlled trial with the objective of evaluating whether patients with SUI treated with MS have a higher continence rate than sham-treated patients.

### Aims of the study

The aim of the study is to investigate the effects of MS in female patients with SUI. Furthermore, we intend to show that MS therapy is safe and effective when provided in a community setting and to prove its overall cost-effectiveness. The following research questions were developed:

- What is the efficacy of MS in female patients with SUI?
- Does the use of MS lead to a better QoL in patients with SUI?
- Is the use of MS cost-effective compared with the conventional therapy?
- After the first 2 months of treatment:
  - How effective is active MS in patients who did not respond to the initial sham treatment?
  - Does longer treatment with active MS improve treatment response in the initial active group?

## Methods/Design

### Study design

**Fig. 1:**
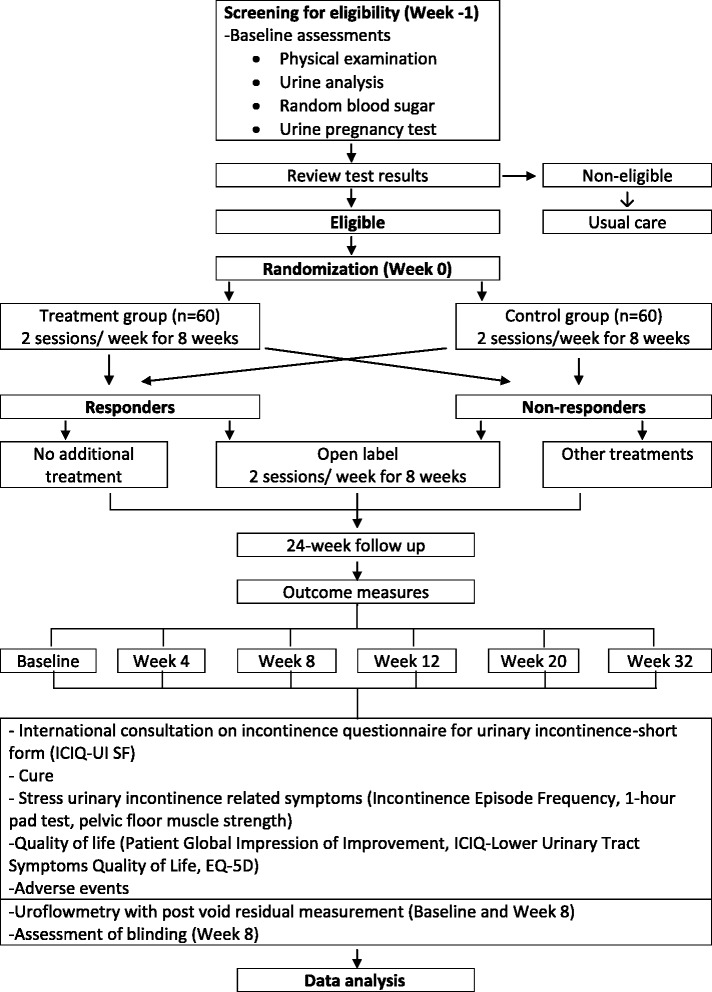
Trial flowchart

This is a multicenter, randomized, double-blinded, sham-controlled, parallel-group trial in a 1:1 allocation ratio. Potential subjects will be screened from among patients attending urology or obstetrics and gynecology clinics of the participating hospitals in Northern Malaysia: Island Hospital, Lam Wah Ee Hospital, Penang Adventist Hospital, Pantai Hospital, Metro Specialist Hospital and Loh Guan Lye Hospital, as well as from among the general population through advertisements in posters, brochures, newspapers and websites. The diagnosis of UI will be made by either the urologist or the gynecologist [1]. Methods, definitions and units conform to the standards jointly recommended by the International Continence Society and the International Urogynecological Association, except where specifically noted [1]. The flowchart and study design schedule are presented in Fig. 1 and Table 1, respectively.

**Table 1:** Study design schedule

	Screening and baseline	Treatments					Follow–up		
Visit	0	1	2–7	8	9–15	16	17	18	19
Timeline (wk)	0	1	1–4	4	5–8	8	12	20	32
Screening for eligibility	x								
Informed consent	x								
Demographic data and medical history	x								
Physical examination	x								
Urine analysis	x								
Random blood sugar	x								
UPT	x					x			
Uroflowmetry with postvoid residual (ultrasound)	x					x			
Randomization		x							
MS treatment		x	x	x	x	x			
Adverse events		x	x	x	x	x	x	x	x
Retreatment or new intervention							x	x	x
ICIQ-UI-SF	x			x		x	x	x	x
Incontinence episode diary	x			x		x	x	x	x
1-hr pad test	x			x		x	x	x	x
PFMF (perineometer)	x			x		x	x	x	x
PGI-I				x		x	x	x	x
ICIQ-LUTS-QoL	x			x		x	x	x	x
EQ-5D	x			x		x	x	x	x
Blinding assessment						x			

*Abbreviations: ICIQ-LUTS-QoL* International Consultation on Incontinence Questionnaire–Lower Urinary Tract Symptoms Quality of Life, *ICIQ-UI-SF* International Consultation on Incontinence Questionnaire for Urinary Incontinence Short Form, *MS* magnetic stimulation, *PFMF* pelvic floor muscle function, *PGI-I* Patient Global Impression of Improvement, *UPT* urine pregnancy test

### Study population

#### Inclusion criteria

To be eligible for participation, subjects are required to fulfill the following conditions: (1) female aged 21 years and older, (2) demonstration of urine leak on coughing at a bladder volume of approximately 200 to 250 ml, (3) International Consultation on Incontinence Questionnaire for Urinary Incontinence Short Form (ICIQ-UI-SF) score of at least 6 points (range: 0–21), (4) must be able and agree to carry out 1-hour pad test and (5) voluntary participation and signing of the informed consent form. Urodynamic testing will not be performed as an eligibility criterion [21].

#### Exclusion criteria

The following are the exclusion criteria: (1) patients with urgency UI, mixed UI or overflow UI; (2) acute severe infections (e.g., pneumonia); (3) severe cardiac arrhythmia; (4) cardiac pacemaker or other implanted metallic devices; (5) neurologic conditions (e.g., stroke, epilepsy, Parkinson disease, multiple sclerosis); (6) random blood sugar above 10 mmol/L; (7) pregnant or actively trying to conceive; (8) previous surgery for SUI; (9) pelvic or gynecological surgery for less than 3 weeks or in the next 8 weeks; (10) previous treatment with MS; (11) history of pelvic irradiation; (12) concurrent medications with α-adrenergic antagonists (e.g., terazosin, tamsulosin, doxazosin), diuretics, serotonin-norepinephrine reuptake inhibitors or any other medications known to worsen incontinence; (13) stage III or IV pelvic organ prolapse according to Pelvic Organ Prolapse Quantification System [22]; (14) severe urethral sphincter weakness and/or defect; (15) suspected urethral and/or vesical fistula; (16) urinary tract infection or hematuria; and/or (17) postvoid residual volume greater than 200 ml.

#### Additional restrictions

Subjects of childbearing age who are not actively conceiving are required to be on any effective contraception methods, including (1) established use of oral, injected or implanted hormonal methods of contraception; (2) placement of an intrauterine device or intrauterine system; (3) barrier methods of contraception, including condom or occlusive cap (diaphragm or cervical or vault caps) with spermicidal foam, gel, film, cream or suppository; (4) male sterilization (with the appropriate postvasectomy documentation of the absence of sperm in the ejaculate); and (5) true abstinence.

Subjects who are taking α-adrenergic antagonists or any other medications known to worsen incontinence will be recruited into the study only if the medications can be discontinued. If the medications can be discontinued, the subjects will undergo a 14-day washout period and then be reassessed for eligibility.

Subjects are prohibited from starting additional specific treatments for SUI, including PFMT, biofeedback, vaginal cone, electrical stimulation or pharmacological treatments such as duloxetine.

### Interventions

All subjects will be provided with counseling on lifestyle modification, including (1) appropriate fluid consumption (between 2 and 2.5 L/24 hr or approximately 250 ml/2 hr), (2) weight reduction if body mass index is 30 or higher and (3) caffeine reduction (limited to one to two cups per day).

**Fig. 2:**
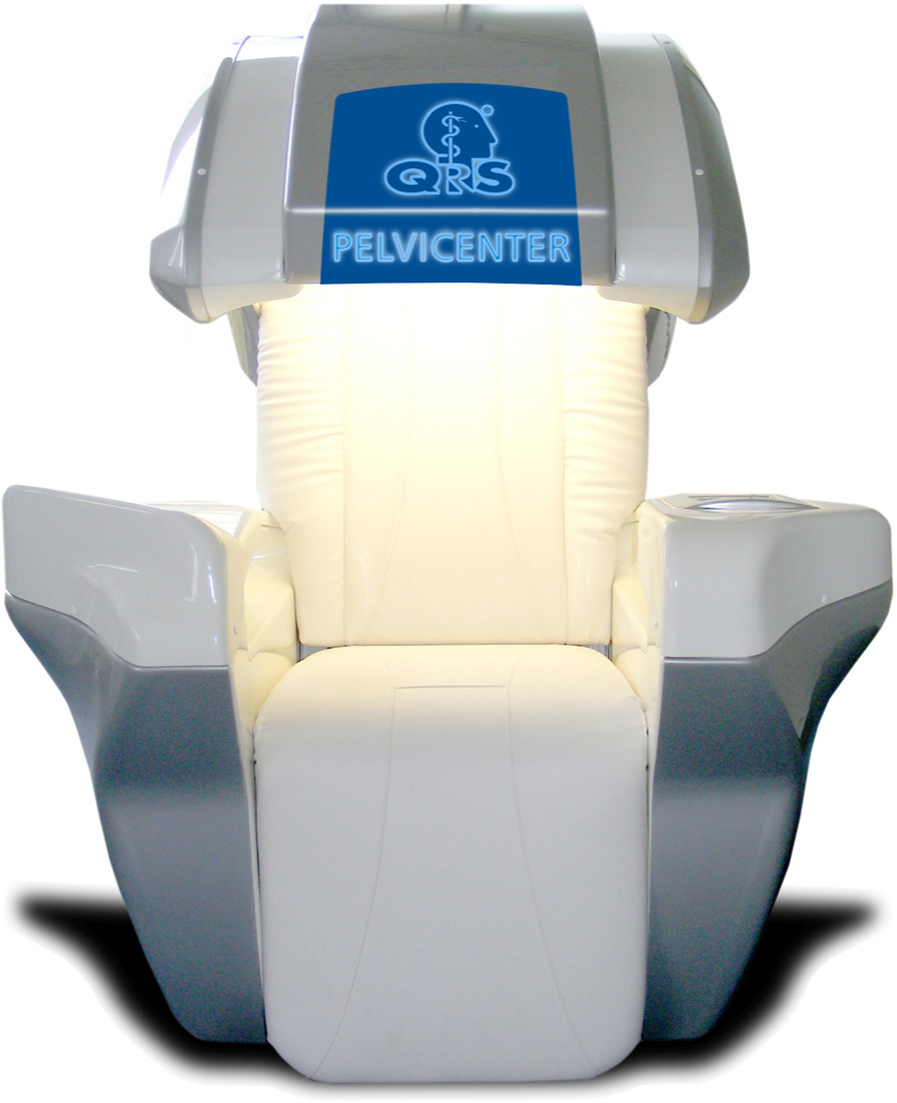
QRS-1010 PelviCenter magnetic stimulation device

The intervention will involve the use of QRS-1010 PelviCenter (QRS International, Ruggell, Liechtenstein) (Fig. 2). This medical device uses electromagnetic pulsing technology for pelvic floor muscle stimulation, which was approved as a conservative treatment for UI by the U.S. Food and Drug Administration in June 1998 [11] and by the European Commission in January 2011. It generates a homogeneous magnetic field via a magnetic coil embedded beneath the surface of the seat. When applied with high electric currents, the magnetic coil generates pulsed electromagnetic fields that are able to penetrate deep into the pelvic floor to reach the relevant conductive tissues. The changing magnetic field induces a flow of ions to propagate electrical eddy currents. A voltage gradient ensues, and membrane depolarization occurs. During this consistent trajectory, a sufficiently large membrane depolarization will result in an action potential along the nerve tissues. In the pelvic floor, this leads to pelvic floor nerve stimulation (stimulation of motor end plates) and ultimately pelvic floor muscle contraction [11, 23].

In both groups, treatment involves two sessions of 20 minutes each per week for 8 weeks (16 sessions total). After the 8-week treatment, subjects who are not satisfied with their treatment outcome will be given the opportunity to enroll in an open-label phase, regardless of their treatment allocation. Subjects will be considered treatment defaulters if they miss treatments for more than 2 consecutive weeks.

#### Active magnetic stimulation

Subjects will be seated with the perineum in the middle of the seat to allow for the greatest effect of the pulsing magnetic field on the pelvic floor and sphincter muscles. Stimulation intensity will be gradually increased, beginning with 20 % simulation on the first session, followed by increments of 20 % (or maximum tolerable stimulation) until they receive stimuli at 100 % intensity using a stimulation repetition cycle of 50 Hz in an 8-s “on” 4-s “off” pulsing manner by a study nurse not involved in the assessments.

#### Sham magnetic stimulation

**Fig. 3:**
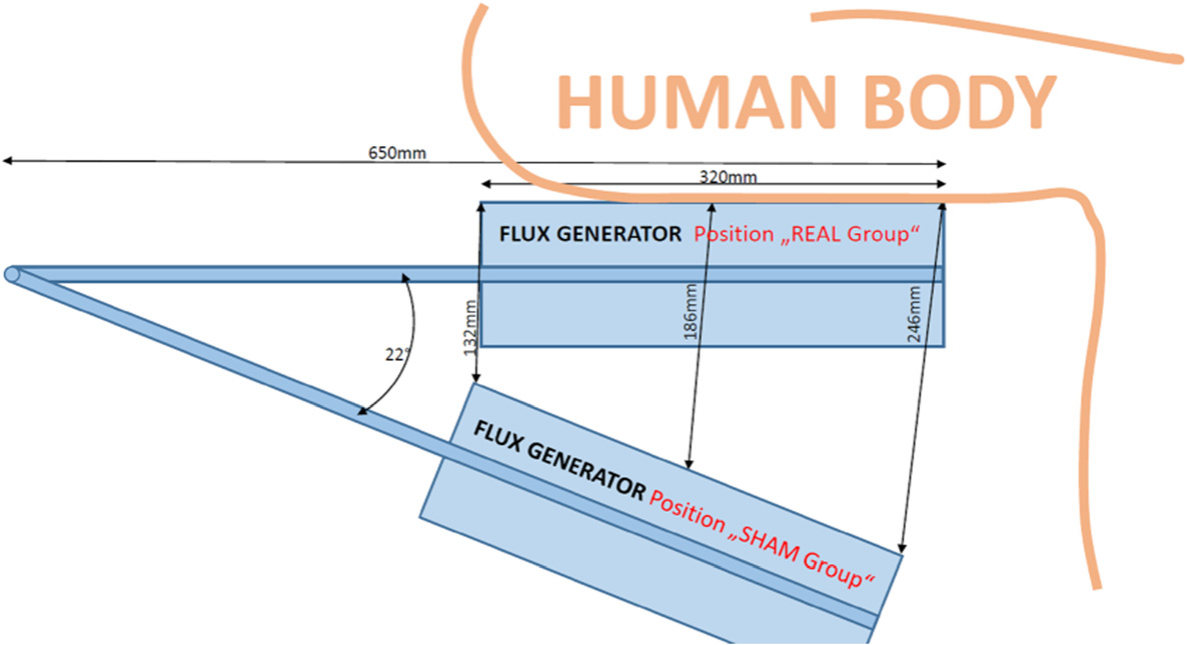
Flux generator positions of active versus sham stimulation

To ensure that sham subjects’ experiences are similar to the intervention, the sham group will undergo the same number, duration and frequency of treatments as the active group using the same MS device and stimulation frequency. However, unlike the intervention, the magnetic coil will be tilted to 22° down with a lower stimulation intensity, beginning with 20 % simulation intensity at the first session, followed by gradually increasing increments of 20 % intensity after every five sessions until a maximum of 60 % intensity is reached (Fig. 3). The combination of these methods has been shown to provide a total energy output of 136 kJ, (or 8.500 J each session) during the 8-week study period, which is far less than the energy output of one 20-minute active mode run (at 100 % intensity) of 408 kJ, thus providing a similar appearance as the active treatment.

### Study outcomes

#### Primary outcome

The primary outcome measure is the improvement in the severity of involuntary urine loss. This is measured by the self-administered ICIQ-UI-SF, which measures frequency, volume and impact on daily life of involuntary urine loss. The ICIQ-UI-SF has undergone extensive psychometric testing and is graded A (highly recommended by the International Consultation of Incontinence) as an outcome measure in research trials [24]. The primary criterion for response is defined as having a 5-point or greater reduction in the ICIQ-UI-SF score (score range: 0–21) from baseline to 8 weeks [25–27]. The responder criterion of at least a 5-point decrease will allow detection of clinically significant improvement in SUI symptoms.

#### Secondary outcomes

1. Cure
  As the ultimate aim of treatment is to achieve complete continence, this is measured through a combination of an objective and a subjective outcome measure. *Cure* is defined as a leakage of less than 1 g on the pad test [28] or a “never” response to question 3 of the ICIQ-UI-SF, “How often do you leak urine?”
2. SUI-related symptoms
  - Incontinence episode frequency (IEF)
    Symptoms relevant to SUI (incontinence episodes and complications) will be measured using a self-completed incontinence diary. The outcome measure is the number of responders, defined as having at least a 50 % reduction in IEF compared with baseline [29]. The average daily incontinence episode is calculated on the basis of a 3-day incontinence episode diary.
  - 1-hour pad test
    The outcome measure is the number of responders, defined as having a decrease of 50 % or more in pad weight compared with baseline [14]. The 1-hour pad test will be conducted according to guidelines published by the International Continence Society [30]. However, the test will be initiated when the bladder volume is at 250 ml (as measured by abdominal ultrasound) instead of drinking 500 ml of liquid. The modification is based on recommendations from the Fifth International Consultation of Incontinence, in which experimental conditions for short-term pad tests must include a standardized bladder volume [28]. Subjects are judged as dry when leakage is less than 1 g on the pad test and as improved when pad weight decreases by 50 % or more [14, 28].
  - Pelvic floor muscle strength
    The outcome measure will be an improvement in the pelvic floor muscle strength compared with baseline as measured using the Peritron perineometer (LABORIE International, Mississauga, ON, Canada) [31, 32]. The Peritron is a pressure-sensitive dynamometer used for objective assessment of the strength of pelvic floor muscle contractions. For each subject, a sterile latex sleeve is fitted around the silicone rubber sheath and inserted into the vagina. After restoring to point 0, the subjects will be asked to perform a maximal pelvic floor contraction. After each contraction, the calibrated 0 point will be restored. The peak, average and duration of contraction for three consecutive contractions will be measured and recorded.
  - Incontinence severity
    Subjects are divided into the following four categories of SUI severity according to the ICIQ-UI-SF score: slight (1–5), moderate (6–12), severe (13–18) and very severe (19–21) [33]. The outcome measure will be the number of responders, defined as at least one level of improvement in severity groups (e.g., from moderate to mild).
3. Quality of life (QoL)
  Another measure of treatment success can be defined as an improvement in the subjects’ QoL. To ensure that these measures are captured and reported, the following questionnaires will be used:
  - Patient Global Impression of Improvement (PGI-I), a single-item generic measure that allows subjects to rate their condition regarding their response to the therapy. The outcome measure is the number of responders, defined as subjects who answer “very much better” and “much better” to the PGI-I question [34].
  - The International Consultation on Incontinence Questionnaire–Lower Urinary Tract Symptoms Quality of Life, which is based on the King’s Health Questionnaire [35], is a condition-specific questionnaire used to evaluate the QoL of UI patients. The possible score ranges from 19 to 76, with higher values indicating increased impact on QoL. The outcome measure will be an improvement in the QoL of subjects compared with baseline [35, 36].
  - The EQ-5D is a simple, generic QoL measure that is able to provide a single index status. This will be subsequently converted into a weighted health state index using the Malaysian value sets.

### Health economic evaluation and analysis

In addition to the evaluation of clinical treatment effectiveness, a prospective assessment of health economic issues is being planned. A cost-effectiveness analysis using patient-reported outcomes will be performed. To estimate costs, the resource use is measured in natural units (e.g., duration of treatment in minutes). The average resource use per patient is calculated and priced with opportunity cost approximated over Malaysian market prices or schedule prices (e.g., wage agreements for staff costs). A sensitivity analysis is performed by replacing the mean costs and effects with the upper and lower bounds of the respective 95 % confidence intervals. Costs and effectiveness results are synthesized in an incremental cost-effectiveness ratio.

### Safety and adverse events

MS has a good safety profile, with no reported adverse effects [37]. Nevertheless, all adverse events, which include (but are not limited to) any unexpected signs and symptoms, newly diagnosed disease, unusual laboratory findings or hospitalizations, will be documented, regardless of whether the adverse event is related to MS. In cases where there is deterioration in a subject’s preexisting disease during the course of the study, this information will also be documented as an adverse event. During every follow-up visit, subjects will be asked if they are experiencing any adverse effects.

### Randomization

Subjects will be assigned in a 1:1 allocation to either the active or sham MS group using computer-generated, permuted block randomization, with variable block sizes of four, six and eight, by an independent pharmacist with no clinical involvement in the trial. Details of the allocated group are given in sequentially numbered, opaque, sealed envelopes to an appointed nurse involved in administering the treatments to subjects. The independent pharmacist who generates the randomization list will ensure that the envelopes are opaque and impermeable when held to the light. Additionally, the sealed envelopes are stored in a locked cabinet accessible only by the appointed nurse. Enrollment of subjects (assessment of eligibility, discussion of the trial, obtaining informed consent) will be done by investigators and clinicians who are blinded to treatment allocation. Following enrollment of a subject, the investigator will assign the subject a unique identification number. In the first treatment session for each subject, the appointed study nurses will sequentially open the envelopes. The subject’s unique identification number and date will be written on the appropriate allocated envelope.

### Blinding and unblinding and control for bias

Investigators who carry out outcome measure assessments and statisticians who perform data analysis will not be informed of the allocation assignment. The same appointed study nurses who sequentially open the treatment allocation envelopes will be in charge of administering treatments for the subjects during each visit, and they will not be involved in any outcome assessments. Formal training on operating the QRS-1010 PelviCenter will be provided to the study nurses by an engineer from QRS International. Additionally, treatment appointments will be handled by the independent study nurses, and the investigators will not come into contact with the subjects at any time during the treatment sessions. All personnel involved in the study will adhere to standard operating procedures, with clear separation between investigators who conduct outcome assessments and study nurses who administer the treatments.

In the event of an unexpected medical emergency, subject allocation may be unblinded to the investigator or clinician. The investigator will report the unblinding to the sponsor, followed by submission of a detailed report to the sponsor. The events in which unblinding may occur include (but are not limited to) (1) the subject becomes pregnant while receiving the intervention; (2) an adverse event requires unblinding to provide safety information necessary to manage the subject’s condition; and (3) a serious adverse event requires unblinding to decide whether to continue or discontinue participation. An unblinding report will be completed by the investigator, with reasons for the unblinding specified.

Various measures will be taken to control for sources of bias. For selection bias, differences between the baseline characteristics of the two treatment groups will be compared to ensure that randomization is accomplished successfully. The appointed study nurse will sequentially open the envelopes, which will be counterchecked by another study nurse to ensure correct implementation of treatment assignments. To avoid performance and detection bias, the investigators are not allowed to be present in the treatment room. The appointed nurses who administer treatment will not be involved in enrolling the patients or in any outcome measure assessments. To evaluate the effectiveness of blinding of study participants, each subject will be asked to answer the following question at the end of the 16 treatment sessions: Which treatment do you think you received? The subject will have three possible answers: “active treatment”, “don’t know” or “sham treatment”. Patients’ ability to correctly identify treatment assignment at the end of the 8-week treatment will be analyzed and compared between the two treatment groups. Finally, to control for attrition bias, differences between the number of withdrawals from the study, including the reasons for withdrawal, will be analyzed and compared between the two treatment groups.

### Subject withdrawal

The subjects may choose to withdraw from the study, or they may be withdrawn from the study, at any time at the discretion of the investigator. If a subject withdraws or is withdrawn, every effort will be made to complete and report the observations as thoroughly as possible.

The possible reasons for withdrawal include (but are not limited to) (1) subject’s decision, (2) intolerable adverse events, (3) clinically significant laboratory abnormality, (4) protocol violation (e.g., incorrectly enrolled or randomized), (5) subject requires use of unacceptable concomitant medication, (6) subject not compliant with protocol procedures, (7) subject develops a condition during the study that violates the inclusion and exclusion criteria (e.g., pregnancy), (8) death and/or (9) any other reason, in the investigator’s opinion, that would impede the subject’s participation in the study.

In the event of pregnancy, the subject will be monitored until the conclusion of the pregnancy, and the outcome of the pregnancy will be reported. If a subject ends her participation in the study before randomization (before the first treatment visit), she will be replaced by another randomly selected patient. If this happens, the situation and circumstances will be documented in the case report form.

### Statistical methods

#### Sample size calculation

Two types of sample size calculations were performed: non-repeated measures and repeated measures. Our primary outcome of interest is the difference in proportion of responders between the active and sham groups after 8 weeks of treatment, defined as a reduction of 5 points or more from baseline to 8 weeks in the ICIQ-UI-SF total score. On the basis of outcomes derived from previously published data on MS, it is anticipated that MS will improve symptoms in 60 % (p1 = 0.6) of subjects with SUI, whereas sham MS will improve symptoms in 30 % (p2 = 0.3) of subjects with SUI [11, 13]. Thus, a minimum sample size of 88 (44 per arm) is powered to detect a difference of at least 30 % (δ = 0.3), with a 95 % confidence interval (α = 0.05) and statistical power of 80 % (β = 0.2) [38]. Allowing for a 25 % dropout rate, the enrollment goal is 120 subjects.

We also calculated our sample size using PASS^13^ Sample Size Software (NCSS Statistical Software, Kaysville, UT, USA) [39]. The tests for two proportions in a repeated-measures design, which can be used to calculate sample size in a mixed-model analysis of repeated measures data, was selected from among the PASS^13^ statistical tools. Using the same parameters (δ = 0.3, α = 0.05 and β = 0.2), an autoregressive AR(1) covariance pattern with autocorrelation of 1.0, and a design with six repeated measurements, a total sample size of 84 (*n* = 42 in each arm) was obtained. As the sample size calculated using a test of simple differences in proportion was higher, we selected the initial calculated sample size.

#### Statistical analysis

Data entry will be performed using Excel 2007 software (Microsoft, Redmond, WA, USA). IBM SPSS Statistics for Windows version 21.0 software (Armonk, NY, USA) will be used to analyze the collected data. All statistical analysis will be conducted by an independent statistician, with blinding of assessors maintained. A *p*-value <0.05 is considered statistically significant.

Descriptive statistics will be used to analyze baseline characteristics. To compare baseline demographic data between the treatment groups, independent *t*-tests (or the Mann–Whitney *U* test) will be used to analyze continuous data, and χ^2^ or Fisher’s exact tests will be used for categorical data.

In the main analysis, we will compare all subjects who are randomized in this study on an intention-to-treat basis, irrespective of whether they completed the allocated treatments. Data will be analyzed by using a longitudinal method (repeated-measures design). No missing data imputation will be implemented (e.g., mean imputation, last observation carried forward, multiple imputation). Instead, a linear mixed model (LMM) and a generalized linear mixed model (GLMM) that do not require formal imputation methods will be used [40]. Continuous dependent variables will be analyzed with LMM, fitted with restricted maximum likelihood estimation [41]. The multilevel approach (LMM and GLMM) will be used, as it accounts for the non-independence among observations in repeated-measures data and controls for the effect of individuals by including extra parameters to include any random effects [42]. Furthermore, a different covariance matrix can be specified, providing enhanced flexibility for longitudinal data analysis, as opposed to the traditional repeated-measures analysis of variance (ANOVA), which assumes compound symmetry (i.e., equal variances and covariances over time) [43]. Compound symmetry means that the pattern of covariances or correlations is constant across trials, which can be an unrealistic assumption. Mauchly’s test of sphericity in repeated-measures ANOVA is used to test whether sphericity (a less stringent form of compound symmetry) is met. Repeated-measures ANOVA must meet various assumptions, including compound symmetry, balanced data and complete dataset to ensure robust use.

The main outcome measure (responder analysis of ICIQ-UI-SF, dichotomous variable) and other dichotomous variables will be analyzed using GLMM (binomial logistic regression). We will estimate the marginal means of proportions, odds ratios comparing treatments and the corresponding 95 % confidence intervals. The fixed effects include time, code and interactions between code and time. Code comprises two binary coded variables, with the sham group set as the reference group. We are primarily interested in the code and time interaction, which represents the difference in outcome variables between groups.

For responder analysis of outcome measures, subjects who withdraw after randomization (missing data) will be considered as treatment failures and will be included in the denominator. The responder analysis between treatment groups will be compared using GLMM (binary logistic regression).

### Quality control and monitoring

Before commencing the trial, the protocol will be reviewed and revised by all parties involved in the study, including urologists, gynecologists and academicians. All investigators and nurses involved in the clinical trial will be trained on the study procedures and will attend the Good Clinical Practice workshop before the start of the trial. The current curriculum vitae and training records of all investigators will be forwarded to the sponsor and the ethics committee.

To maximize subject retention, timely reminders for appointments via telephone calls or text messages will be sent by study investigators. Subjects who discontinue treatments will be encouraged to return for follow-up. Nevertheless, in the event of dropout or subject refusal of follow-up, the subject’s decision will be acknowledged.

In accordance with the requirements of International Conference on Harmonization Good Clinical Practice, regular monitoring of the study will be carried out throughout the study. The investigator accepts a monitoring representative from the sponsor to visit the clinical trial centers regularly to ensure that the key requirements of the study are met. The monitoring representative will ascertain that the correct course of clinical trial study is conducted and the case report forms are accurate, reliable and complete.

To ensure patient confidentiality, all case report forms and relevant documents will be kept in locked cabinets. Each subject will be identified by a unique subject number. All files will be kept separately from identifying information used for subject tracking and follow-up contacts. The results of the research will not become part of the subject’s medical history and will be used only for study purposes. The data published in reports, publications or presentations will not disclose any identifying information.

Any adverse events that are encountered will be recorded in the case report form. Any serious adverse events that occur during the course of the clinical trial will be documented in the case report form and reported within 24 hours of first knowledge of the event to the sponsor and the ethics committee.

In the event of any injury resulting from the research procedures, the sponsor assumes liability by law on behalf of the investigators to compensate the cost of medical treatment provided to the subjects. Financial compensation is not available from the sponsor. The cost of medical care of any illness or injury not directly related to subjects’ participation in the study will not be compensated. An insurance policy is provided by the sponsor of the study.

### Ethics and dissemination

The study will be conducted in accordance with the International Committee of Harmonization guidelines for Good Clinical Practice and the Declaration of Helsinki, and it has been approved by the Joint Ethics Committee of the School of Pharmaceutical Sciences, USM-Hospital Lam Wah Ee on Clinical Studies [USM-HLWE/IEC/2013(0006)]. Written informed consent will be obtained from each subject by the investigators before the subject enters the trial.

During the course of the study, if important protocol modifications are planned, amendments will be submitted to the ethics committee for reapproval. A new written consent form will be given to the subjects if the changes concern them (such as changes in the duration of treatment, changes in inclusion or exclusion criteria).

Only the investigators will have access to the final trial dataset. The sponsor will have access to the final trial dataset after all statistical analyses have been performed. To disseminate our findings, the clinical trial results will be published in peer-reviewed journals.

## Discussion

Our trial is a prospective, randomized, double-blind, sham-controlled trial of SUI using pulsed MS. The trial is adequately powered and will use validated outcomes. Previous studies conducted with MS had inherent flaws, resulting in inconclusive evidence on this treatment modality. A search of trial registers indicates that there are currently no ongoing, prospective clinical trials investigating our research question of interest (last search done on 5 December 2014).

There is a need for a high-quality randomized clinical trial on this topic with a sham group, adequate sample size, appropriate methodology and validated outcome measures, as well as long-term follow-up [19]. We intend to address the shortcomings of previous studies in our trial and to obtain convincing evidence on the efficacy of MS. We acknowledge the importance of applying a sham MS as a control. We will use a specially designed MS device for the purposes of this trial. The appearance and noise are identical to the active treatment. The sum of energy output of the 8-week sham treatment is lower than the sum of energy output of one 20-minute active mode run (K Dobler, personal communication). Furthermore, we will recruit only subjects who are naive to MS. All subjects will be told that they may experience a tingling sensation. Subjects will also be assessed for effectiveness of blinding at the end of the 8-week treatments. To further allow for double-blinding, all assessors involved in the study will be deliberately prohibited from entering the treatment room, and treatment will be coordinated by independent study nurses. Collectively, these measures allow better blinding of subjects with the aim to diminish the placebo effects.

Next, we determined our sample size based on a priori calculation to ensure that our study is adequately powered to answer our primary outcome of interest. To evaluate the effects of MS on SUI, we selected various subjective and objective outcome measures as recommended by international guidelines [44, 45]. Further, we chose a patient-reported outcome, the ICIQ-UI-SF questionnaire, as our primary outcome measure because we recognize the importance of judging treatment benefit from a patient’s perspective [24]. We hope that use of standardized, internationally accepted tools will ease data pooling in meta-analyses, limiting the number of high-quality clinical trials required to draw concrete conclusion on this treatment option.

MS is a non-invasive procedure and has been shown to be effective in SUI in various open-label studies [23]. Further advantages are no reported adverse events, unnecessary to undress, automatic contractions and no pain. If the efficacy of MS is better than PFMT, which has a success rate of approximately 30 %, it may be a reasonable option for patients with SUI who are not keen to undergo surgery. The results of our trial are expected to add essential confirmatory data regarding whether MS is effective for women with SUI.

Our protocol has some limitations. To maximally exclude any placebo effects, various considerations were taken when designing the sham MS. We used the same MS device, designed a coil that can be tilted to adjust the strength of contractions, and included only subjects who are naive to MS. Nevertheless, we think that our blinding methods may be unsuccessful in some patients. Selective refusal by participants in the present study may limit the generalizability of the study results to the wider public. Furthermore, when we first designed the trial, we specified a follow-up period of 6 months for logistical reasons. However, we intend to prolong our follow-up period to a minimum of 1 year, as recommended by guidelines [45]. Amendments have been made and submitted to our local ethics committee. Finally, we selected our sample size based on our primary outcome data analysis method (responder analysis: percentage of subjects who meet primary criterion of response). Our study may be underpowered for determination of statistical significance for the secondary outcome measures.

### Trial status

The trial has completed its recruitment but is continuing to follow-up patients.

## Additional file

Additional file 1:
**Summary of published magnetic stimulation clinical trials on stress urinary incontinence.** The table provides a summary of previous studies conducted worldwide that were designed to test the effects of magnetic stimulation on stress urinary incontinence. The design of the trial, number of subjects, treatment protocol (including intensity and frequency) and results are presented.

## Abbreviations

ANOVA: Analysis of variance
GLMM: Generalized linear mixed model
ICIQ-LUTS-QoL: International Consultation on Incontinence Questionnaire–Lower Urinary Tract Symptoms Quality of Life
ICIQ-UI-SF: International Consultation on Incontinence Questionnaire for Urinary Incontinence Short Form
IEF: Incontinence episode frequency
LMM: Linear mixed model
MS: Magnetic stimulation
PFMF: Pelvic floor muscle function
PFMT: Pelvic floor muscle training
PGI-I: Patient Global Impression of Improvement
QoL: Quality of life
SUI: Stress urinary incontinence
UI: Urinary incontinence
UPT: Urine pregnancy test
