# Supplementary material for: Magnetic stimulation for stress urinary incontinence: study protocol for a randomized controlled trial
Source: Trials. 2015 Jun 21;16:279. doi: 10.1186/s13063-015-0803-1 (PMC4477591; doi:10.1186/s13063-015-0803-1)
Supplement: Additional file 1: — Summary of published magnetic stimulation clinical trials on stress urinary incontinence. The table provides a summary of previous studies conducted worldwide that were designed to test the effects of magnetic stimulation on stress urinary incontinence. The design of the trial, number of subjects, treatment protocol (including intensity and frequency) and results are presented. [file 13063_2015_803_MOESM1_ESM.docx]

**Additional file 1. Summary of published magnetic stimulation clinical trial on stress urinary incontinence**

| **Author, Year, Country** | **Design** | **Subject (Active: Sham)** | **Age (SD)** | **Treatment protocol** | **Intensity** | **Frequency** | **Control** | **Follow-up** | **Results** |
| --- | --- | --- | --- | --- | --- | --- | --- | --- | --- |
| Galloway, 1999, USA [[11](#_ENREF_11)] | Prospective open-label | 50 | 55 | Twice a week for 6 weeks | NA | 10 minutes 5Hz, 1-5 minutes rest, 10 minutes 50Hz | None | >3 months | At 3 months,  -17 (34%) were dry  -16 (32%) were using not more than 1 pad/day  -17 (34%) were using >1 pad/day |
| Galloway, 2000, USA [[12](#_ENREF_12)] | Prospective open-label | 47 | 55 (13) | Twice a week for 6 weeks | NA | 10 minutes 5Hz, 1-5 minutes rest, 10 minutes 50Hz | None | 6 months | At 6 months,  -13 (28%) were dry  -25 (53%) were using no pad or <1 pad/day  -Pad use decreased in 33 (70%). -Median number of pads decreased from 2.16 to 1 per day (P<0.005) -Frequency of leak decreased from 3.0 to 1.7 (P= 0.004) |
| Fujishiro, 2000, Japan [[13](#_ENREF_13)] | Randomized controlled trial | 62 (31:31) | 58 | Once only, 5 seconds/ minute for 30 minutes | 50% intensity of maximum output | 15Hz | Sham stimulation with inactive device | 1 week | One week after treatment, improvement and cure rates:  -Active group: 74% (n=23) and 13% (n=4) versus sham group: 32% (n=10) and 3% (n=1) (p=0.0009) |
| Yamanishi, 2000, Japan [[14](#_ENREF_14)] | Prospective open-label | 7 | 63.4 (10.3) | Twice a week for 5 weeks | NA | 15 minutes 20Hz, 1 minute on 30 seconds off | NA | None | -2 (28.6%) cured  -4 (57.1%) improved |
| Unsal, 2003, Turkey [[17](#_ENREF_17)] | Prospective open-label | 29 | 55 | Twice a week for 8 weeks | NA | 10 minutes 5 Hz, 10 minutes, 50 Hz | None | 1 year | At 12 months,  - 11 (38%) cured  - 12 (41%) improved |
| Lee, 2004, Korea (abstract) [[16](#_ENREF_16)] | Prospective comparative | 49 (29 BIOCON, 20 Neocontrol) | NA | 20 minutes, two to three times a week for 12 weeks | NA | NA | NA | NA | -Quality of life scored improved from 2.4 to 6.05 in Neocontrol group and 2.21 to 6.03 for the BIOCON group  -No statistical difference when comparing their vaginal pressures |
| Yokoyama, 2004, Japan [[15](#_ENREF_15)] | Prospective open-label | 17 | 60.1 (12.6) | Twice a week for 8 weeks | NA | 10 minutes 10Hz, 2 minutes rest, 10 minutes 50Hz | None | 6 months | At 8 weeks,  - 9 (52.9%) cured  -7 (41.1%) improved.  -3 (17.6%) recurred and received an operation within 24 weeks after the last treatment |
| Manganotti, 2007, Italy [[39](#_ENREF_39)] | Randomized controlled trial | 20 (10:10) | 50.1 (2.86) | 15 minutes, three times a week for 2 weeks | 60% intensity | 15 Hz, 3seconds/ minute | Sham stimulation with inactive device | 1 month | At 1 week after therapy,  -Active stimulation showed improvement in health perception (P<0.001), social limitation (P<0.01), sleep/energy performance (P<0.05) and severity measure (P<0.05), not seen in sham stimulation  -These results were no longer observed at 1 month after treatment |
| Hoscan, 2008, Turkey [[40](#_ENREF_40)] | Prospective open-label | 27 | 53 | Twice a week for 6 weeks | NA | 10 minutes 5Hz, 1-5 minutes rest, 10 minutes 50Hz (5s on 5s off) | None | 2 years | At 3 months,  -8 (29.7%) cured and 13 (48.1%) improved  Cumulative success rates:  -77.8% at 3 months  -66.6% at 12 months  -40.7% at 24 months |
| Gilling, 2009, New Zealand [[41](#_ENREF_41)] | Randomized controlled trial | 70 (35:35) | Active: 54 (2.0) Sham: 58 (2.2) | Three times a week for 6 weeks | Maximum level  tolerated | 10 minutes 10 Hz, 3 minutes rest, 10 minutes 50 Hz | Thin deflective Aluminium plate inserted in chair | 6 months | At 8 weeks or 6 months,  -The significant improvements in 20-min pad-test, 24-h pad-test, number of pads/day, I-QoL score and KHQ score in active stimulation group were not statistically significant when  compared with the sham-treatment group |
| Ismail, 2009, UK [[42](#_ENREF_42)] | Prospective open-label | 27 | 51 (13) | Twice a week for 8 weeks | NA | 10 minutes 5Hz, 2 minutes rest, 10 minutes 50Hz | NA | 3 months | At end of treatment or at 3 months,  -No significant change in outcome measures  -Side effects in 52.1% of patients and dropout rate was 35.4% |
| Doganay, 2010, Turkey [[43](#_ENREF_43)] | Prospective open-label | 68 | 55.8 (10.3) | Twice a week for 8 weeks | NA | 10 minutes 5Hz, rest 1-5 minutes, 10 minutes 50Hz, 5s on 5s off | None | 3 years | At 6 months,  - 32 (47%) were dry, 27 (39%) improved in frequency of daily leak episodes from 3.2 times to 1.2 times.  At 3 years,  -Symptoms deteriorate gradually and close to baseline. |
| Bakar, 2011, Turkey [[18](#_ENREF_18)] | Prospective open-label | 13 | 65.23 (2.8) | Twice a week for 6 weeks | NA | 10 minutes 5Hz, 5 minutes rest, 10 minutes 50 Hz, 5s on 5s off | None | None | -Pad test reduction, 4.31g ± 6.75 to 1.51g ± 1.73 (p = 0.016) -EMG improved, 373.77 ± 138.17 to 503.46 ± 192.93 (p = 0.005)  -I-QoL score improved, 42.76 ± 26.69 to 26.84 ± 20.7, (p = 0.002)  -UDI-6 score improved, 7.38 ± 2.73 to 4.07 ± 2.75 (p = 0.002) -VAS 5.81 ± 2.6 to 2.61 ± 1.98 (p = 0.006) |

EMG: Electromyography, KHQ: King's Health Questionnaire, I-QoL: Urinary Incontinence Quality of Life Scale, NA: Not available, UDI-6: Urogenital Distress Inventory, VAS: Visual Analogue Scale
